# Role of Focal Adhesion Tyrosine Kinases in GPVI-Dependent Platelet Activation and Reactive Oxygen Species Formation

**DOI:** 10.1371/journal.pone.0113679

**Published:** 2014-11-21

**Authors:** Naadiya Carrim, Tony G. Walsh, Alessandra Consonni, Mauro Torti, Michael C. Berndt, Pat Metharom

**Affiliations:** 1 Department of Experimental Medicine, Royal College of Surgeons in Ireland, Dublin, Ireland; 2 Curtin Health Innovation Research Institute, Faculty of Health Sciences, Curtin University, Perth, Australia; 3 Laboratories of Biochemistry, Department of Biology and Biotechnology, University of Pavia, Pavia, Italy; University of Sydney, Australia

## Abstract

**Background:**

We have previously shown the presence of a TRAF4/p47^phox^/Hic5/Pyk2 complex associated with the platelet collagen receptor, GPVI, consistent with a potential role of this complex in GPVI-dependent ROS formation. In other cell systems, NOX-dependent ROS formation is facilitated by Pyk2, which along with its closely related homologue FAK are known to be activated and phosphorylated downstream of ligand binding to GPVI.

**Aims:**

To evaluate the relative roles of Pyk2 and FAK in GPVI-dependent ROS formation and to determine their location within the GPVI signaling pathway.

**Methods and Results:**

Human and mouse washed platelets (from WT or Pyk2 KO mice) were pre-treated with pharmacological inhibitors targeting FAK or Pyk2 (PF-228 and Tyrphostin A9, respectively) and stimulated with the GPVI-specific agonist, CRP. FAK, but not Pyk2, was found to be essential for GPVI-dependent ROS production and aggregation. Subsequent human platelet studies with PF-228 confirmed FAK is essential for GPVI-mediated phosphatidylserine exposure, α-granule secretion (P-selectin (CD62P) surface expression) and integrin α_IIb_β_3_ activation. To determine the precise location of FAK within the GPVI pathway, we analyzed the effect of PF-228 inhibition in CRP-stimulated platelets in conjunction with immunoprecipitation and pulldown analysis to show that FAK is downstream of Lyn, Spleen tyrosine kinase (Syk), PI3-K and Bruton's tyrosine kinase (Btk) and upstream of Rac1, PLCγ2, Ca^2+^ release, PKC, Hic-5, NOX1 and α_IIb_β_3_ activation.

**Conclusion:**

Overall, these data suggest a novel role for FAK in GPVI-dependent ROS formation and platelet activation and elucidate a proximal signaling role for FAK within the GPVI pathway.

## Introduction

Glycoprotein (GP)VI is a major platelet collagen receptor. Following vascular injury, platelet binding to immobilized collagen within the extracellular matrix initiates a cascade of intra-platelet signaling pathways which are essential for platelet activation and subsequent thrombus formation [1]. GPVI ligation initiates an array of platelet responses, including platelet spreading, granule secretion, integrin α_IIb_β_3_-dependent aggregation, and reactive oxygen species (ROS) generation [2], [3]. While previous studies have demonstrated that platelet-derived ROS are associated with collagen-induced thrombus formation, the signaling molecules involved in GPVI-dependent ROS generation remain poorly defined [4]–[8].

We have previously shown the presence of a GPVI-associated complex involving tumor necrosis factor receptor-associated factor (TRAF)4, the NADPH oxidase (NOX) organizer subunit, p47^phox^, Hic5, and proline rich tyrosine kinase 2 (Pyk2), consistent with a potential novel role of this complex in GPVI-dependent ROS formation [9]. Pyk2, a Ca^2+^-dependent, non-receptor protein tyrosine kinase (PTK) and its closely related family member, focal adhesion kinase (FAK), are known to be involved in intracellular ROS-dependent signaling. Pyk2 was recently shown to be a key regulator of NOX-dependent ROS formation in endothelial cells [10]. Importantly, both FAK and Pyk2 are activated downstream of ligand binding to GPVI, but the significance of both these PTKs in GPVI-dependent ROS formation and an extensive characterization of their relevance to the GPVI signaling pathway remains unclear [11], [12].

As the only two known members of the FAK family, FAK (125 kDa) and Pyk2 (110 kDa) share 45% sequence identity. Each contains a C-terminal focal adhesion target (FAT) domain, a catalytic tyrosine kinase, proline-rich regions and a unique N-terminal four-point-one, ezrin, radixin, moesin homology (FERM) domain, which once phosphorylated, allows docking of SH-domain containing proteins such as Src, Fyn, p130cas and the focal contact adaptor proteins, Paxillin, and Hic-5 [13]–[17]. Initial Pyk2 activation through autophosphorylation of Tyr-402 is critical for its function as this leads to the recruitment of Src-family kinases (SFKs) which further phosphorylate Pyk2, elevating its catalytic activity and interaction with other adapter and effector molecules [18]. Similarly, Tyr-397 has been identified as the key autophosphorylation site on FAK which facilitates Src-mediated phosphorylation of Tyr-576 and -577 [19]. In particular, both FAK family members have been implicated as essential regulators of cytoskeletal dynamics, particularly through modulation of the Rho family GTPase members Rac and Rho. They also regulate other important downstream signaling molecules such as phosphoinositide 3-kinase (PI3-K) and phospholipase C (PLC)-γ isoforms [20]–[24].

Studies in recent years have described various functional roles for the FAK family in platelets. While the FAK knockout mouse model is embryonically lethal, Hitchcock *et al.* demonstrated that mice with platelet-specific FAK-deficiency are predisposed to increased tail bleeding times and that their platelets responded poorly to GPVI agonists [25]. Consistently, defects in human GPVI-mediated aggregation, calcium mobilization and dense granule (ATP) secretion have also been reported using the FAK inhibitor, PF-228 [26]. More recently however, comparable effects of PF-228 were reported in FAK deficient platelets in *in vitro* (platelet aggregation) and *in vivo* (carotid occlusion artery) assays relative to wild type mice [27]. Interestingly, studies on Pyk2-deficient platelets demonstrate no significant differences in *in vitro* GPVI-induced platelet responses (aggregation, α-granule secretion and spreading). However, Pyk2-deficient platelets exhibit a marked reduction in thrombus formation over collagen and ablated G-protein-coupled receptor (GPCR)-mediated platelet activation [28], [29]. Furthermore, there is considerable controversy regarding the specific signaling mechanisms regulating activation of FAK family members in platelets. For example, tyrosine phosphorylation of FAK and Pyk2 can occur through integrin-dependent and integrin-independent mechanisms following platelet activation while the relevance of protein kinase C (PKC) to Pyk2 activation is still a matter of debate [12], [30]–[33]. Most notably however, both PTKs can be differentially regulated in platelets, suggesting a potential functional divergence between these two signaling molecules [34].

In this study, we aimed to clarify the relative roles of Pyk2 and FAK in GPVI-dependent platelet activation, with particular emphasis on ROS formation and the localization of these PTKs within the GPVI pathway. We confirmed through pharmacologic and genetic (Pyk2 knockout) inhibitory strategies that FAK, and not Pyk2, is essential for GPVI-dependent ROS formation and other important functional responses such as α-granule secretion (P-selectin), phosphatidylserine (PS) exposure, and integrin activation, while Pyk2 appears to be non-essential with respect to the GPVI pathway. Moreover, we show FAK as a proximal signaling molecule in the GPVI pathway, downstream of Lyn, Spleen tyrosine kinase (Syk), PI3-K and Bruton's tyrosine kinase (Btk) but upstream of Rac1, PLCγ2, Ca^2+^, PKC, Hic-5, NOX1 and α_IIb_β_3_ activation.

## Materials and Methods

### Materials

Anti-FAK, anti-Pyk2, the anti-phosphotyrosine antibody, 4G10, and HRP-conjugated goat anti-mouse and mouse anti-rabbit light chain specific IgGs were all obtained from Millipore (Lake Placid, NJ, USA); normal rabbit and mouse IgGs and RGD peptide were from Santa Cruz (CA, USA), while anti-PLCγ2 and anti-Hic-5 were from Cell Signaling Technology, Inc. (Boston, MA, USA). Anti-Rac1 was from Tebu-Bio (Peterborough, UK). Cross-linked collagen related peptide (CRP) was purchased from Prof. Richard Farndale (Dept of Biochemistry, Cambridge University, UK). The pharmacological inhibitors, PF-573228 (hereafter referred to as PF-228), PP2, Wortmannin, EHT-1864, U73122, GF109302× and the Ca^2+^ chelator, BAPTA, were from Tocris Bioscience (R&D Systems Europe, UK). Tyrphostin A9 was from Calbiochem. ML171 (2-acetylphenothiazine), and BAY61-3606 (hereafter referred to as BAY) were purchased from Sigma Aldrich (St. Louis, MO, USA).

### Preparation of human and mouse washed platelets

Blood collection from drug-free healthy volunteers was approved by the Medical Research Ethics Committee of the Royal College of Surgeons in Ireland (RCSI), ID number REC269, and written informed consent was obtained from all donors prior to phlebotomy. Venous blood was drawn using acid citrate dextrose (ACD-15% v/v) as anticoagulant. In brief, platelet-rich plasma (PRP) was obtained by centrifugation of whole blood at 190 *g* for 20 min without braking. Platelets were isolated from PRP by centrifugation for 8 min at 650 *g* with prostaglandin (PGE_1_ - 1 µM), resuspended and washed (3x) in CGS buffer (123 mM NaCL, 33.3 mM glucose, 14.7 mM trisodium citrate, pH 7.0) containing 1 µM PGE_1_. Platelets were resuspended to the required count in Ca^2+^-free HEPES-Tyrode's buffer (5 mM HEPES, 5.5 mM glucose, 138 mM NaCl, 12 mM NaHCO_3_, 0.49 mM MgCl_2_, 2.6 mM KCL, 0.36 mM NaH_2_PO_4_, pH 7.4). Platelets were rested for at least 30 min at 37°C and supplemented with 1.8 mM CaCl_2_ prior to experimentation.

Pyk2 knockout (KO) platelet studies were conducted at the Dept. of Biology and Biotechnology, University of Pavia, Italy. The procedures involving the use of mice for the experimental work were approved by the Committee on Ethics of Animal Experimentation (Comitato Etico per la Sperimentazione Animale) of the University of Pavia with authorization number n°1/2011 of 8-02-2011. Mouse blood was collected using ACD at a ratio of 1∶15 and prepared as previously described [22], [35]. The generation and characterization of the Pyk2 knockout (KO) mice has also been previously described [36]. Age- and sex-matched wild-type littermates were used as controls.

BTK KO mice were kindly provided by Dr Caroline Jefferies, Royal College of Surgeons in Ireland. Platelet isolation from these mice was prepared as described for Pyk2 KO platelets. Blood collection from CO_2_ terminally-asphyxiated mice was performed under Licence B100/3779 and RCSI Animal Research Ethics Committee approval.

### Platelet aggregation

Platelet aggregation was preformed in a PAP 4-C aggregometer using washed platelets (2.5×10^8^/mL) under constant stirring at 1100 rpm at 37°C. For all inhibitory studies thoughout this study, platelets were preincubated with vehicle control or antagonists for 10 min at 37°C before the addition of agonist.

### Immunoprecipitation and western blot analysis

For detection of tyrosine phosphorylated FAK, Pyk2, Hic-5 and PLCγ2, stimulated platelets (1×10^9^/mL) were lysed in 10× lysis buffer (final concentration; 1% Triton X-100, 20 mM Tris, 5 mM EGTA, pH 7.4) containing complete protease and phosphatase inhibitor cocktail (Thermo Scientific, IL, USA). Lysis proceeded for 30 min on ice, with subsequent clarification (10 min at 16,000 *g*) and pre-clearance with 15 µL Pansorbin (10% cell suspension-Calbiochem) for 1 hour at 4°C. The appropriate antibody/isotype-matched immunoglobulin control (4 µg) was added to the pre-cleared supernatant which was left rotating at 4°C overnight. Then, 25 µL Pansorbin was added to each sample for a 2 hour incubation at 4°C. Antigen-absorbed Pansorbin was harvested (5 min at 3,000 *g*), washed 3 times with 1× lysis buffer and heated to 100°C in 2× SDS sample loading buffer for 10 min. Immunoprecipitated proteins were resolved by SDS 5-20% polyacrylaimde gel electrophoresis, transferred to a PVDF membrane and immunoblotted with appropriate antibodies. Blots were visualized using HRP-conjugated secondary antibodies and enhanced chemiluminescence (SuperSignal West Pico, Thermo Scientific). Quantitative comparisons between bands were performed using scanning densitometry with ImageJ.

### Rac1 activation assay

Following platelet stimulation studies, washed platelets (6×10^8^/sample) were lysed and harvested for analysis of Rac1 GTP using a Rac1 activation assay kit (Tebu-Bio). This ‘pulldown’ assay utilizes a recombinant protein containing the p21-binding domain of PAK1 fused to GST to selectively isolate Rac1 GTP. The assay procedure was performed as per manufactuers instructions.

### Flow cytometry; analysis of platelet ROS production, fibrinogen binding, p-selectin and phosphatidylserine exposure

All flow cytometric analysis was performed on a FACSCanto II and analyzed using FACSDiva software (Becton Dickinson, San Jose, CA, USA).

### Measurement of intracellular ROS

This assay was performed as previously described with some minor modifcations [37]. In brief, washed platelets (2.5×10^8^/mL) in HEPES-Tyrodes (0.1% BSA) were incubated for 30 min at 37°C with 10 µM dihydrodichlorofluorescein diacetate (H_2_DCFDA-Cambridge Bioscience, UK), pre-treated with antagonists then stimulated with 1 µg/mL of CRP for 10 min at 37°C. Samples were diluted 10-fold in HEPES-Tyrodes (0.1% BSA) containing 10 µM H_2_DCFDA and analyzed immediately.

### Fibrinogen binding assay

Washed platelets (2.5×10^8^/mL) were mixed with Oregon Green 488 conjugated-fibrinogen (Biosciences, Ireland) for 10 min at 37°C. CRP (1 µg/mL) was added and incubated for a further 10 min at 37°C. Reactions were diluted in 900 µL HEPES-Tyrodes (0.1% BSA) and analyzed.

### PS and p-selectin measurements

To measure platelet pro-coagulant capacity and α-granule release, washed platelets (2.5×10^8^/mL) in HEPES-Tyrodes (0.1% BSA) were pre-treated with antagonists and detection reagents; FITC-labeled Annexin V to detect PS exposure and PE-labeled mouse anti-human CD62P/mouse IgG1 Isotype (BD Biosciences, UK), then stimulated with 1 µg/mL CRP for 10 min. Reactions were diluted in 900 µL HEPES-Tyrodes (0.1% BSA) and analyzed.

### Mitochondrial potential/uncoupling assay

To test for non-specific inhibitor-mediated alterations in mitochondrial potential, platelets at (2.5×10^8^/mL) were incubated with the cationic, fluorescent JC-1 dye (Merck), final concentration of 1 µg/mL (15 min at 37°C in the dark). Antagonists were then added for 5 min and the reaction was terminated by the addition of HEPES-Tyrodes. Mitochondrial uncoupling was measured by a decrease in the red/green fluorescence intensity ratio by flow cytometry.

### Data analysis

All statistical analysis was performed using GraphPad Prism 5. Results are shown as mean ± SEM. Statistical significance of difference between means was determined using ANOVA, with post-hoc analysis by the Bonferroni test. A value of *p≤0.05 was considered to be statistically significant.

## Results

### FAK, but not Pyk2, is a prerequisite for GPVI-mediated ROS generation and platelet aggregation

**Figure 1:**
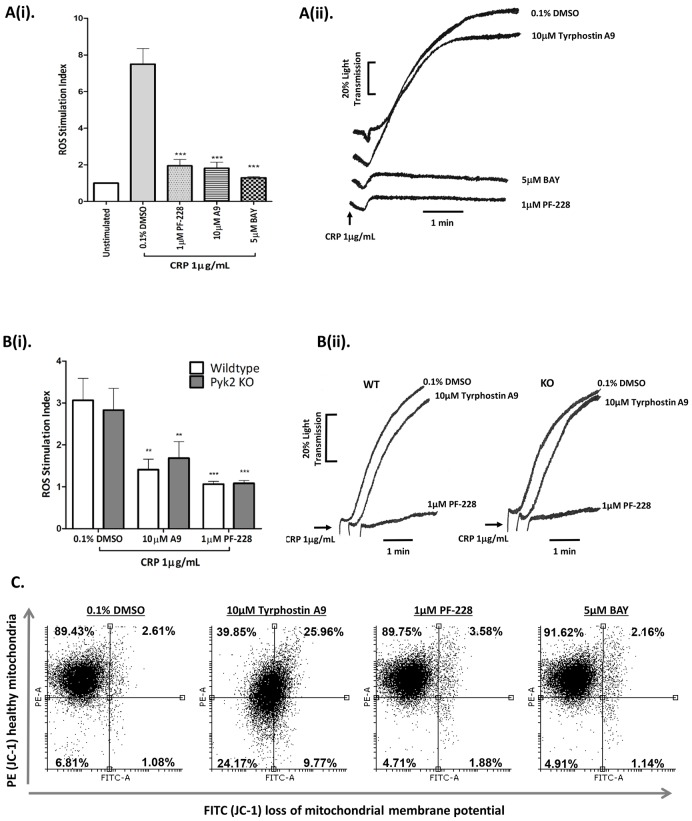
FAK, but not Pyk2 is required for GPVI-mediated ROS generation and platelet aggregation. A. Washed human platelets (2.5×10^8^/mL) preloaded with 10 µM H_2_DCFDA (for ROS experiments only) were pre-treated with vehicle control (0.1% DMSO), FAK inhibitor (1 µM PF-228), Pyk2 inhibitor (10 µM Tyrphostin A9) or Syk inhibitor (5 µM BAY), then stimulated with 1 µg/mL CRP and monitored for ROS generation (i) and platelet aggregation (ii). Data are mean ± SEM (n = 6), ***P≤0.0001 *vs*. 0.1% DMSO. **B.** Similarly, washed platelets (2.5×10^8^/mL) from wild type and Pyk2 knockout mice were pre-treated with 0.1% DMSO, 1 µM PF-228 or 10 µM Tyrphostin A9 and assessed for ROS generation (i) and platelet aggregation (ii) following stimulation with 1 µg/mL CRP. Data are mean ± SEM (n = 6), **P≤0.01, ***P≤0.001 *vs.* 0.1% v/v DMSO. Aggregation traces in A and B are representative of 6 and 4 independent experiments, respectively. **C.** To test for off-target inhibitor effects, washed platelets (2.5×10^8^/mL) treated with vehicle control (0.1% DMSO), 10 µM Tyrphostin A9, 1 µM PF-228 or 5 µM BAY, were pre-loaded with the mitochondrial membrane-permeant JC-1 dye to monitor alterations in the membrane potential of the mitochondria. Changes in potential, which are based on a decrease in red (PE)/green (FITC) intensity ratios were quantified by flow cytometry. Results are representative of three independent experiments.

To examine the role of FAK and Pyk2 in GPVI-dependent platelet activation, human platelets were pre-treated with the FAK inhibitor, PF-228, and Pyk2 inhibitor, Tyrphostin A9, and monitored for CRP-induced ROS production and aggregation. The Syk-specific inhibitor, BAY, was used as a positive control, as it has been previously shown to block CRP-induced ROS formation [37]. Inhibitory response curves of Tyrphostin A9 and PF-228 to CRP-induced ROS are demonstrated in Figure S1. Interestingly, both FAK and Pyk2 inhibition significantly reduced CRP-induced ROS formation, but only FAK was required for platelet aggregation (Figure 1Ai-ii). Tyrphostin A9 has been previously described as a selective Pyk2 inhibitor [38]–[41]. To confirm this, the inhibitor was tested for its effect in Pyk2 deficient platelets, which have normal expression levels of FAK [28]. Interestingly, washed platelets from both wild type (WT) and Pyk2 knockout (KO) mice produced comparable levels of ROS (and platelet aggregation) following stimulation with CRP, while pre-treatment with Tyrphostin A9 completely blocked ROS production in both genotypes, demonstrating that the inhibitory effects of Tyrphostin A9 (10 µM) was due to off-target effects, and more importantly, that Pyk2 did not appear to have a functional role in either ROS production or platelet aggregation in response to GPVI activation (Figure 1Bi–ii). Inhibitors were tested in a cell-free superoxide anion (O_2_^.−^) assay to ensure they had no ROS-scavenging capacity (Figure S2). Further investigation confirmed that Tyrphostin A9 acted as a mitochondrial uncoupler, possibly explaining its indirect effect on ROS production (Figure 1C). In contrast, FAK inhibition was equally effective at blocking CRP-dependent ROS production and platelet aggregation in both WT and Pyk2 KO, suggesting a crucial role for this signaling molecule in GPVI-mediated platelet activation. Importantly, 1 µM PF-228 (or 5 µM BAY) did not alter the platelet mitochondrial membrane potential (Figure 1C).

### GPVI-dependent α-granule release, PS exposure and fibrinogen binding require FAK

**Figure 2:**
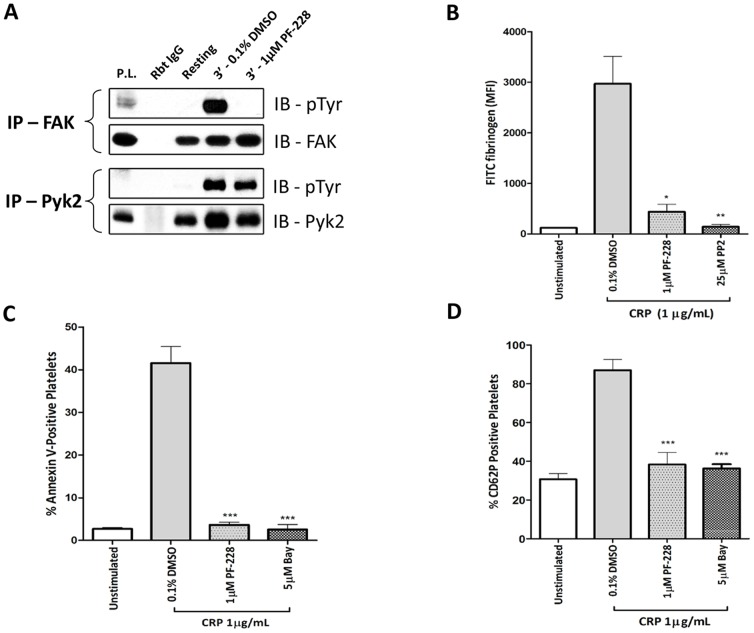
GPVI-dependent α-granule release, PS exposure and fibrinogen binding require FAK. **A.** Washed human platelets pre-treated with vehicle control (0.1% DMSO) or FAK inhibitor (1 µM PF-228) were stimulated with 1 µg/mL CRP for 3 min (with stirring), immunoprecipated with either anti-FAK or anti-Pyk2 and blotted for phosphotyrosine (4G10). Blots are representative of three independent experiments. P.L., platelet lysate; IB, immunoblot. Washed human platelets pre-treated with 0.1% DMSO or PF-228 (1 µM) were stimulated with CRP (1 µg/mL) and measured by flow cytometry for: (**B**) binding of Oregon Green-conjugated fibrinogen, (**C**) phosphatidylserine exposure using FITC-Annexin V and (**D**) α-granule secretion by P-selectin (CD62P) surface expression using PE-labeled anti-CD62P antibody or isotype control. Data are mean ± SEM (n = 3), **P≤0.01, ***P≤0.001 *vs.* 0.1% v/v DMSO.

To confirm FAK inhibitor specificity, washed human platelets that were pre-treated with PF-228 (1 µM) and stimulated with CRP (1 µg/mL), were subjected to immunoprecipitation and phosphotyrosine analysis of FAK and Pyk2. As expected, 1 µM PF-228 blocked tyrosine phosphorylation of FAK but not Pyk2 (Figure 2A). However, as reported by Slack-Davis *et al.* 2007 [42], we confirmed that in platelets, 10 µM PF-228 had off-target effects as it blocked GPVI-mediated Pyk2 tyrosine phosphorylation (Figure S3). Previous studies have implicated key roles for FAK with regards to GPVI-dependent platelet aggregation and spreading [25], [26]. Here, we extended these findings to other readouts of GPVI-mediated platelet activation. Fibrinogen binding, a sensitive indicator of integrin inside-out activation, was significantly blocked by PF-228 following GPVI stimulation (Figure 2B). CRP-stimulated platelets also showed a marked reduction of surface PS (Annexin V binding) and P-selectin (CD62P) following incubation with PF-228 (Figure 2C, 2D). Consistent with a previous study, CRP-mediated secretion of ATP from dense granules was also significantly blocked by FAK inhibition (data not shown) [26]. Together, these results demonstrate that FAK contributes significantly to GPVI-induced integrin activation, PS exposure, alpha and dense granule release, in addition to platelet aggregation, spreading and ROS formation.

### GPVI-dependent FAK activation and ROS production is α_IIb_β_3_-independent

**Figure 3:**
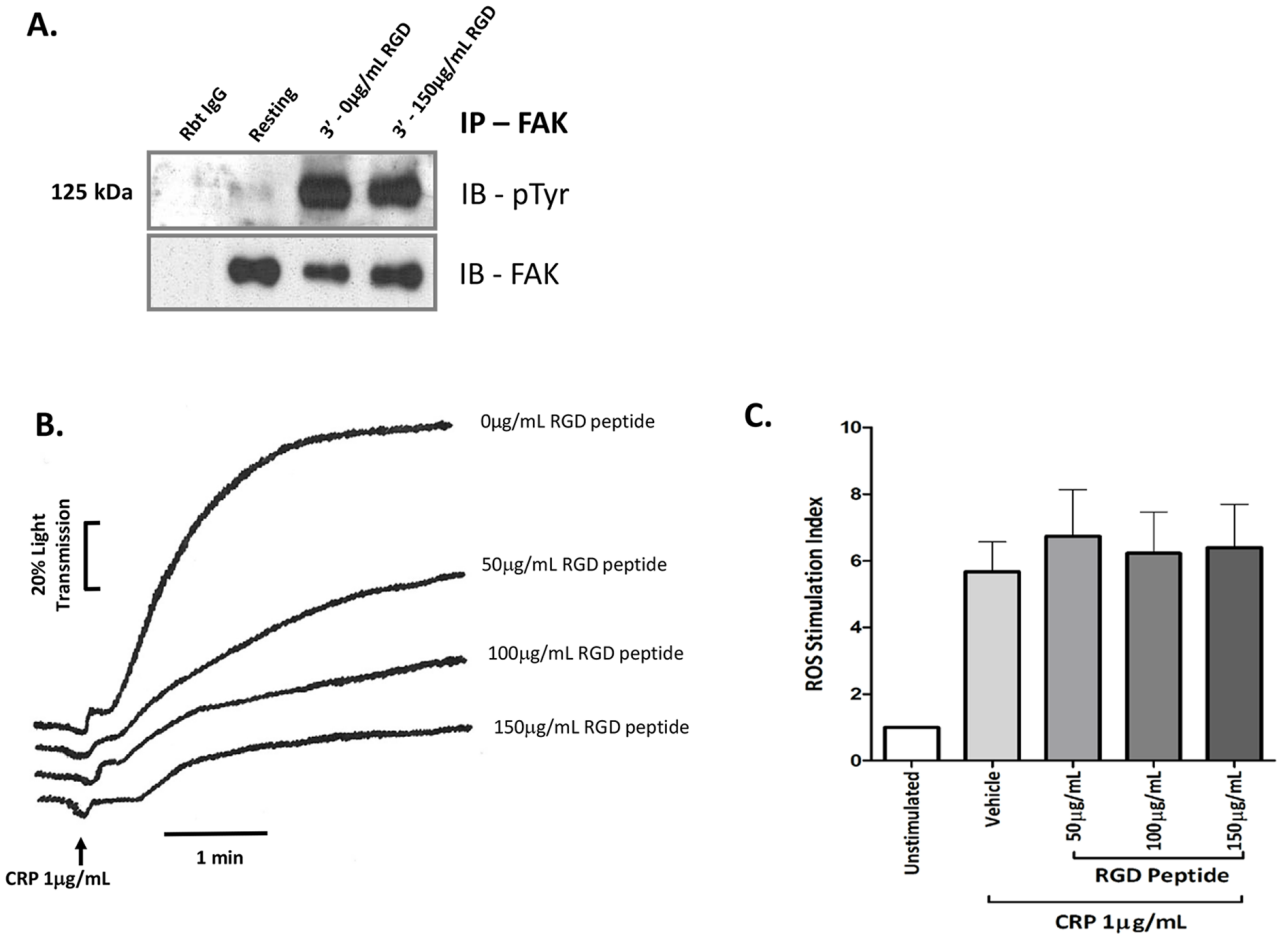
GPVI-dependent ROS production and FAK activation are α_IIb_β_3_-independent. **A.** Washed platelets (1×10^9^/mL) were pre-incubated with RGD peptide (150 µg/mL) and then stimulated with 1 µg/mL CRP for 3 min (with stirring), lysed, immunoprecipitated with anti-FAK (4 µg), analyzed by SDS 5–20% polyacrylamide gel electrophoresis, and immunoblotted for phosphotyrosine with 4G10 or for FAK. Blots are representative of three independent experiments. IB, immunoblot. **B–C.** Washed platelets (2.5×10^8^/mL) pre-treated with various concentrations of RGD peptide (50–150 µg/mL) were stimulated with 1 µg/mL CRP and monitored for (B) platelet aggregation and (C) ROS generation. Aggregation traces are representative of three independent experiments.

Previous studies in platelets are unclear with regard to the role of FAK on integrin α_IIb_β_3_-dependent ‘outside in signaling’ [30], [43]. The extent to which GPVI agonist-dependent FAK phosphorylation depends on integrin activation was investigated by pre-treating platelets with an inhibitor of integrin-ligand interaction, arginine-glycine-aspartic acid (RGD) peptide. The RGD peptide decreased CRP-induced platelet aggregation in a dose-dependent manner but had no effect on FAK phosphorylation or ROS formation, suggesting GPVI-derived ROS and FAK activation are independent of integrin α_IIb_β_3_ activation (Figure 3A–C).

### FAK activation within the GPVI pathway

**Figure 4:**
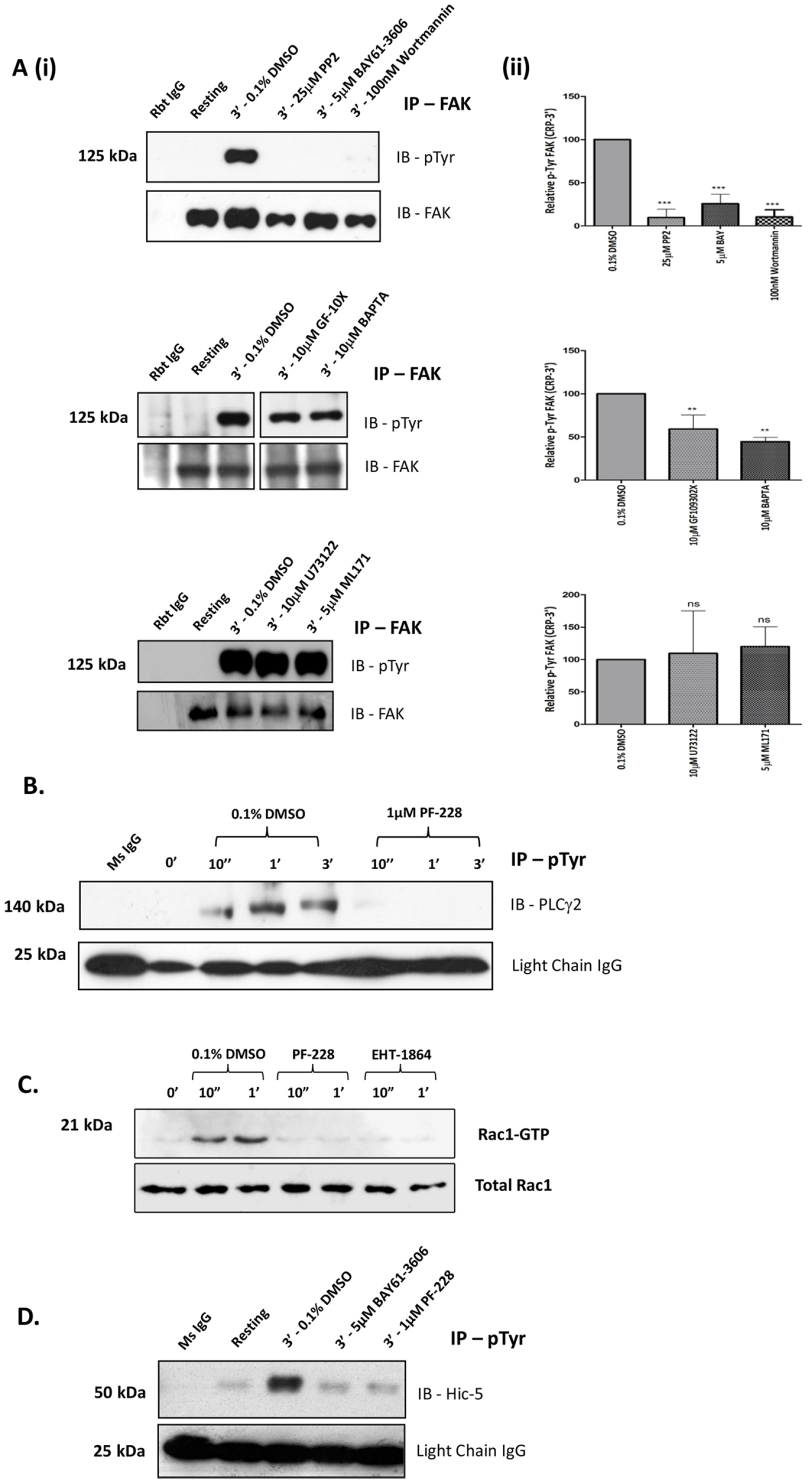
FAK activation within the GPVI pathway. **A(i–ii).** Washed human platelets pre-incubated with vehicle control (0.1% DMSO) or inhibitors; 25 µM PP2, 5 µM BAY, 100 nM Wortmannin, 10 µM BAPTA, 10 µM U73122, 10 µM GF109302X and 5 µM ML171 were stimulated with 1 µg/mL CRP for 3 min (with stirring), lysed, immunoprecipitated with anti-FAK and immunoblotted for phosphotyrosine (4G10) and FAK. Representative blots (Ai) and gel densitometry (Aii) presented as Relative pTyr FAK (i.e. pTyr FAK/total FAK) are shown. Data are mean ± SEM (n = 3), (ns) non-significant, **P≤0.01, ***P≤0.001 *vs.* 0.1% v/v DMSO **B.** Washed platelets pre-incubated with 0.1% DMSO and PF-228 (1 µM), were stimulated with 1 µg/mL CRP for up to 3 min (with stirring), lysed, immunoprecipitated with anti-phosphotyrosine (4G10) and immunoblotted for PLCγ2. Blots are representative of three independent experiments. **C.** Washed platelets pre-incubated with 0.1% DMSO, PF-228 (1 µM) and Rac-1 inhibitor, EHT-1864 (50 µM), were stimulated with 1 µg/mL CRP for up to 1 min (with stirring), lysed, subjected to Rac1 GTP ‘pulldown’ analysis and immunoblotted for Rac1 to detect active ‘GTP’ loaded Rac1. Loading controls for total Rac1 levels were subsequently performed using equal sample volumes. **D.** Washed platelets pre-treated with 0.1% DMSO, BAY (5 µM-included as control) and PF-228 (1 µM) were stimulated with 1 µg/mL CRP for 3 min, lysed, immunoprecipated with anti-phosphotyrosine (4G10) and immunoblotted for Hic-5. IB, immunoblot. Blots are representative of three independent experiments.

Following GPVI stimulation, one of the earliest signaling events to occur is the activation of Syk by the SFKs, Lyn and Fyn, which initiates a well characterized LAT signalosome [44]. To investigate the location of FAK within the GPVI pathway, we monitored CRP-mediated FAK phosphorylation in the presence of pharmacological inhibitors against SFKs (PP2), Syk (BAY), and PI3-K (Wortmannin). These inhibitors significantly suppressed FAK tyrosine phosphorylation following GPVI activation (Figure 4Ai–ii). Furthermore, FAK tyrosine phosphorylation was also downstream of Btk by analysis of WT and Btk KO mouse platelets (Figure S4). While Pyk2 has been described as a Ca^2+^-dependent kinase, we demonstrated that FAK tyrosine phosphorylation following CRP stimulation is partially Ca^2+^-dependent as the intracellular Ca^2+^ chelator, BAPTA (10 µM), partially reduced FAK tyrosine phosphorylation by approximately 50% (Figure 4Ai–ii). Similarly, PKC inhibition with the generic inhibitor, GF109320X, significantly reduced FAK tyrosine phosphorylation. In other cell types FAK has been shown to regulate PLC activation [24]. Consistent with these reports, PF-228 significantly blocked CRP-induced PLCγ2 tyrosine phosphorylation (Figure 4B). In contrast, the PLC inhibitor, U73122, did not inhibit FAK tyrosine phosphorylation (Figure 4Ai–ii). To further establish a link between FAK and GPVI-dependent ROS formation, the effect of PF-228 on Rac1 and Hic-5 activation was analyzed. Rac1 is an essential precursor in the assemblage of an active NOX complex, while Hic-5, through association with TRAF4 has been implicated in oxidant-mediated migration in endothelial cells [45]. Activation of both signaling molecules following CRP stimulation was completely abolished with PF-228 (Figure 4C and D). Further, there was no detectable decrease in CRP-mediated FAK tyrosine phosphorylation in the presence of the NOX1-specific inhibitor, ML171, suggesting FAK activation precedes NOX1 complex formation (Figure 4Ai–ii).

## Discussion

In this paper, we have investigated the role of the FAK family kinases, Pyk2 and FAK, in GPVI-dependent ROS production and platelet activation. Our findings demonstrate that FAK, but not Pyk2, is the crucial PTK regulating GPVI-dependent ROS generation, as well as α-granule secretion, integrin α_IIb_β_3_ activation and PS exposure. Despite Pyk2 phosphorylation occurring after GPVI ligation, there was no functional difference observed with Pyk2 inhibition or deficiency.

Initial experiments in human platelets using the FAK inhibitor, PF-228, and the putative Pyk2 inhibitor, Tyrphostin A9, suggested that both FAK family members were required for GPVI-dependent ROS production, while only FAK was essential for platelet aggregation. However, studies using Pyk2-deficient mouse platelets indicated that the inhibition by Tyrphostin A9 was off target as both WT and Pyk2 KO platelets displayed similar levels of ROS generation and both genotypes were equivalently inhibited by Tyrphostin A9. This result was surprising considering that in platelets Pyk2 is in complex with p47^phox^, a subunit of the NOX2 complex, and additionally that endothelial cells deficient in Pyk2 lack ROS-mediated pro-inflammatory reactions [9], [10]. We have however previously demonstrated that a NOX1-specific inhibitor, ML171, blocks ROS generation from CRP-activated platelets [35]. It is therefore possible that Pyk2 (and the NOX2 complex) are not directly involved in GPVI-mediated platelet activation. Importantly, PF-228 significantly inhibited GPVI-mediated ROS production (and platelet aggregation) in WT and Pyk2 KO platelets, suggesting a fundamental role for FAK in the GPVI pathway controlling ROS production. We confirmed that Tyrphostin A9, but not PF-228, caused mitochondrial membrane depolarization; an effect which decreases cellular ATP levels and may therefore perturb the signaling mechanisms necessary to induce NOX-mediated ROS production following GPVI ligation [46].

To date, FAK is well described for its regulatory role in platelet spreading and more recently for a key role in platelet aggregation, dense granule secretion and Ca^2+^ mobilization following GPVI stimulation [25], [26]. We therefore further characterized the function of FAK in the GPVI signaling pathway and established a regulatory role for this PTK in regards to PS exposure, α-granule secretion and integrin activation. Contrary to evidence in the literature suggesting FAK is an integrin α_IIb_β_3_-dependent kinase, our findings demonstrate that the α_IIb_β_3_-blocking RGD peptide, which inhibited CRP-induced platelet aggregation, did not affect FAK phosphorylation, consistent with the finding that FAK regulates GPVI-induced fibrinogen binding (and aggregation) and is upstream of integrin α_IIb_β_3_-dependent signaling [30]. Further, we found that GPVI-dependent ROS formation was also α_IIb_β_3_-independent, which is in agreement with a recent study that employed platelet-rich plasma [37]. Interestingly, the temporal activation profile of Pyk2 was also integrin α_IIb_β_3_-independent (data not shown), implicating similar regulatory mechanisms for both PTKs in the GPVI pathway, contrary to the differential regulation observed in VWF-stimulated platelets [34].

**Figure 5:**
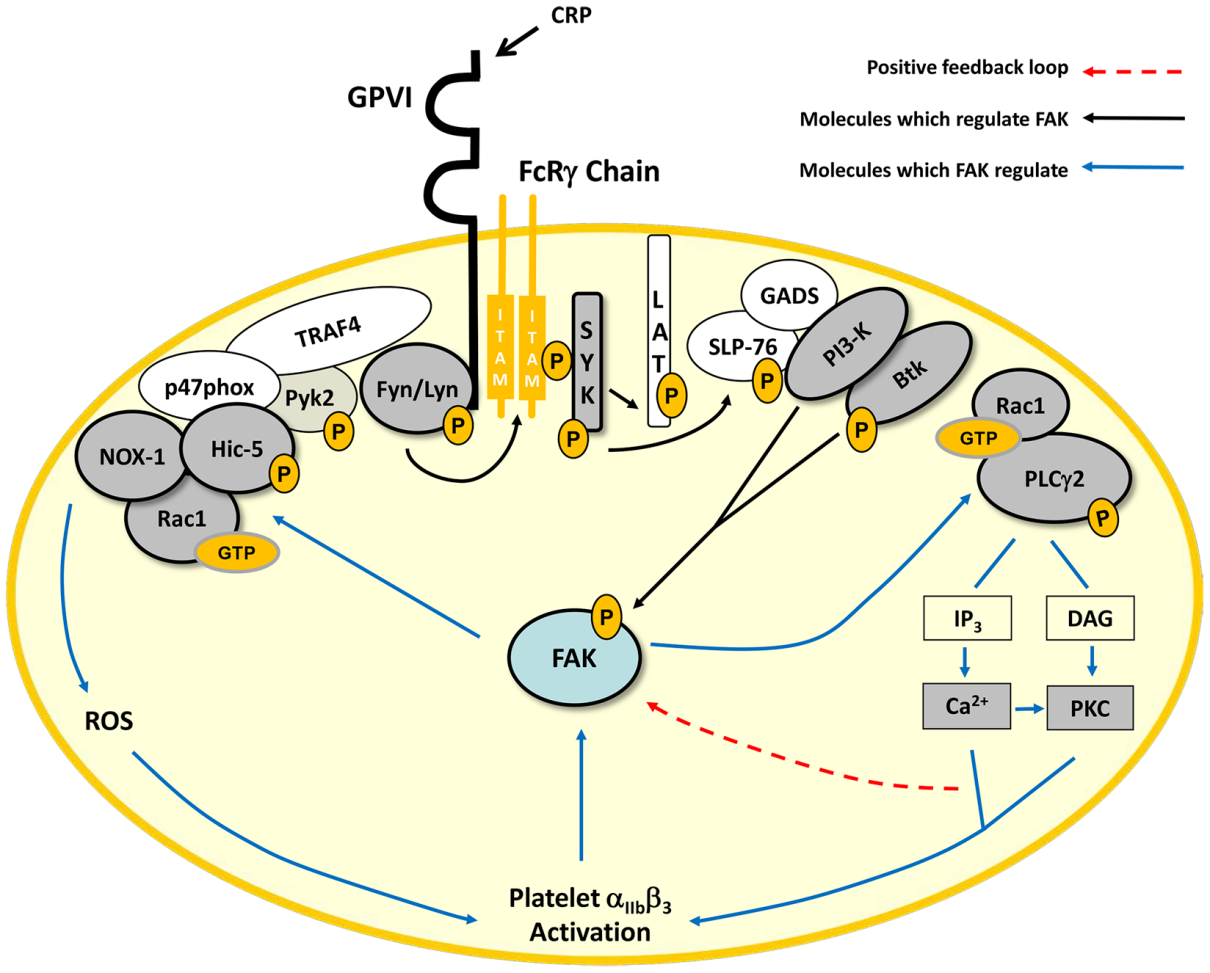
Location of FAK in the GPVI pathway. GPVI activation with CRP triggers FcRγ-chain ITAM phosphorylation through Fyn and Lyn, which recruits and activates Syk, initiating a LAT signalosome involving LAT, SLP-76, Gads, PI3-K, Btk, Rac1 and PLCγ2. FAK is downstream of SFKs, Syk, PI3-K and Btk but upstream of Rac1 and PLCγ2 following GPVI ligation. Activated PLCγ2 produces secondary messengers, IP3 and DAG that induce Ca^2+^ mobilization and PKC activation, respectively, allowing FAK activation and integrin α_IIb_β_3_ engagement, which is also known to regulate FAK activity. FAK also regulates the NOX1 modulator, Rac1, which along with Hic-5, leads to the activation of NOX1, resulting in ROS generation. Key proteins investigated in this study are shaded.

GPVI signals through an immuno tyrosine-based activating motif (ITAM) mediated pathway. Phosphorylation of ITAM sequences within the non-covalently associated FcRγ-chain by SFKs, Lyn and Fyn, allows assemblage of Syk and subsequent activation of a well characterized Linker for Activation of T-cells signalosome involving Src homology (SH)-2 domain-containing leukocyte phosphoprotein of 76 kDa (SLP-76), PI3-K, Btk, Rac1 and PLCγ2, which facilitate calcium mobilization and PKC activation (Figure 5) [47]–[50]. To investigate the regulation of FAK within this pathway, we adopted a pharmacological and genetic approach targeting a number of these key signaling molecules. Pharmacological inhibition of SFKs, Syk and PI3-K completely blocked CRP-induced FAK activation. Consistent with this, Jones *et al*. reported that PF-228 did not affect CRP-induced Syk phosphorylation [26]. Interestingly, a previous study demonstrated that thrombin-mediated PI3-K activity required FAK, highlighting the difference between different agonist signaling pathways [23].

Recruitment of Btk to the plasma membrane is regulated through its pleckstrin homology domain, which binds the PI3-K product, phosphatidylinositol 3,4,5-triphosphate (PIP_3_). We found, using Btk deficient mouse platelets, that GPVI-mediated FAK phosphorylation is downstream of Btk activation. In contrast, PLCγ2 activation was shown to be FAK-dependent as the generic PLC inhibitor, U73122, did not block CRP-induced FAK phosphorylation and conversely, PF-228 inhibited PLCγ2 phosphorylation. FAK has been shown to bind the γ1 isoform of PLC in fibroblasts, an interaction mediated by the SH2 domain of PLCγ1 and Tyr-397 on FAK [24]. While a regulatory role for FAK in GPVI-dependent PLCγ2 activation was demonstrated, we could not detect a physical association between the two signaling proteins in immunoprecipitation/western blot experiments (data not shown). Interestingly, Ca^2+^ chelation and PKC inhibition reduced GPVI-induced FAK phosphorylation. In particular, the finding using BAPTA is of interest as it in part conflicts with the observation that PF-228 inhibits Ca^2+^ mobilization. However, our findings support the literature that Ca^2+^ and PKC are known intermediaries of FAK activation and thus suggest the potential of a positive feedback loop for FAK activation following Ca^2+^ mobilization and PKC activation (Figure 5) [30].

The RhoGTPase, Rac1, is a critical component in the GPVI pathway and has been shown to regulate PLCγ2 [51]. Consistent with studies in other cell types, we found that GPVI-dependent Rac1 activation was downstream of FAK, consistent with the observation that activation of the Rac1 effector, p21 activated kinase (PAK1), is FAK-dependent following GPVI stimulation [20], [26], [52]. NOX1-mediated ROS generation has also been shown to be regulated by Rac1, providing further evidence for a potential regulatory role of FAK in NOX-mediated ROS production [53]. Consistently, NOX1 inhibition did not affect GPVI-mediated FAK activation; in addition, the redox-and GPVI-associated signaling molecule, Hic-5, was regulated by FAK confirming that FAK activation precedes NOX-mediated ROS production.

We recently showed that blocking of platelet ROS production does not significantly reduce CRP-induced platelet activation (aggregation, integrin αIIbβ3 activation, α- and dense granule secretion) [35]. However, thromboxane A2 production and collagen-induced thrombus formation is reduced by blocking ROS production, suggesting divergent, distal signaling roles for ROS in the context of GPVI-mediated platelet activation. Notably, blocking of early GPVI signaling events (i.e. Lyn, Syk, and PI3K) inhibits both platelet activation and CRP-induced ROS formation (unpublished data for Lyn and PI3K), suggesting proximal GPVI signaling events are critical for both. Similarly, our experimental data in figure 4 indicate a close association between FAK activation and early GPVI signaling events, which may explain the defect in both CRP-induced platelet activation and ROS production in the presence of FAK inhibitor PF-228. Importantly, pre-treatment with the NOX1 inhibitor, ML171, did not alter GPVI-mediated FAK phosphorylation, confirming FAK is upstream of platelet ROS production (Figure 4).

Notably, during the preparation of this paper, Roh *et al.* reported off-target effects for PF-228 [27]. They found comparable aggregation responses between WT and FAK-deficient platelets in response to physiological agonists, which were inhibited in the presence of 1 µM PF-228 in both phenotypes. Interestingly, FAK^-/-^ platelets have been previously demonstrated to show defective spreading in response to CRP, but CRP-induced platelet aggregation was not explored in the Roh *et al.* study. Consistent with previous studies documenting compensatory roles for Pyk2 in FAK^-/-^ cells, the authors speculate that compensatory changes in Pyk2 expression and phosphorylation may account for the dispensable phenotype in particular functional assays [54], [55]. It is also unclear whether the off-target effect of PF-228 seen in mouse platelets is relevant in human platelets. In our hands, 1 µM PF-228 does not inhibit GPVI-dependent Pyk2 phosphorylation in human platelets, although it is possible that in the absence of FAK, PF-228 could target Pyk2, its closest family member, as it binds to the ATP binding pockets of these tyrosine kinases [56]. However, the paper which characterized PF-228 as a FAK inhibitor only refers to off target drug effects in cell-based assays at concentrations of 10 µM, but not at 1 µM [42]. Furthermore, our data with inhibitors of key platelet signaling proteins illustrate that phosphorylation of FAK as an integral part in GPVI-dependent ROS generation in platelets.

In summary, our study describes a novel role for FAK in GPVI-mediated ROS formation and demonstrates a key role for FAK in the GPVI signaling pathway, which precedes Rac1, PLCγ2, NOX1 and integrin α_IIb_β_3_ activation. Despite being GPVI-regulated, Pyk2 appears to be dispensable for these functional outcomes. We therefore demonstrate for the first time key functional differences between these two closely related FAK family members following GPVI pathway activation.

## Supporting Information

Figure S1 **Washed human platelets (2.5×10^8^/mL) preloaded with 10 µM H_2_DCFDA were pre-treated with vehicle control (0.1% DMSO), Pyk2 inhibitor (0.5–25 µM Tyrphostin A9) or FAK inhibitor (0.1–5 µM PF-228), then stimulated with 1 µg/mL CRP and monitored for ROS generation.** Data are plotted in Graphpad Prism as stimulation index vs log [inhibitor] µM to determine the inhibitor IC50 values.
(TIF)

Figure S2 **FAK and Pyk2 inhibitors do not scavenge superoxide anion.** Using a cell-free superoxide anion (O_2_^.−^) assay, pharmacological inhibitors PF-228 (1 µM), Tyrphostin A9 (10 µM) and BAY (5 µM) were tested for the capacity to scavenge ROS (N-acetylcysteine was included as positive control). Data are mean ± SEM (n = 3), **p≤0.01 *vs.* DMSO.
(TIF)

Figure S3 **Washed human platelets pre-treated with vehicle control (0.1% DMSO) or FAK inhibitor (PF-228) at 1 µM or 10 µM were stimulated with 1 µg/mL CRP for 3 min (with stirring), immunoprecipated with anti-Pyk2 and blotted for phosphotyrosine (4G10).** Blots are representative of three independent experiments.
(TIF)

Figure S4 **Washed platelets (1×10^9^/mL) from wildtype or Btk knockout mice were stimulated with 1 µg/mL CRP for 3 min (with stirring), lysed, immunoprecipitated with anti-FAK (4 µg), then analysed by SDS 5–20% polyacrylamide gel electrophoresis and immunoblotted for phosphotyrosine (4G10) and FAK.** Blots are representative of two independent experiments.
(TIF)

Methods S1 (DOCX)
