# Supplementary figures and images for: Role of Focal Adhesion Tyrosine Kinases in GPVI-Dependent Platelet Activation and Reactive Oxygen Species Formation
Source: PLoS One. 2014 Nov 21;9(11):e113679. doi: 10.1371/journal.pone.0113679 (PMC4240642; doi:10.1371/journal.pone.0113679)

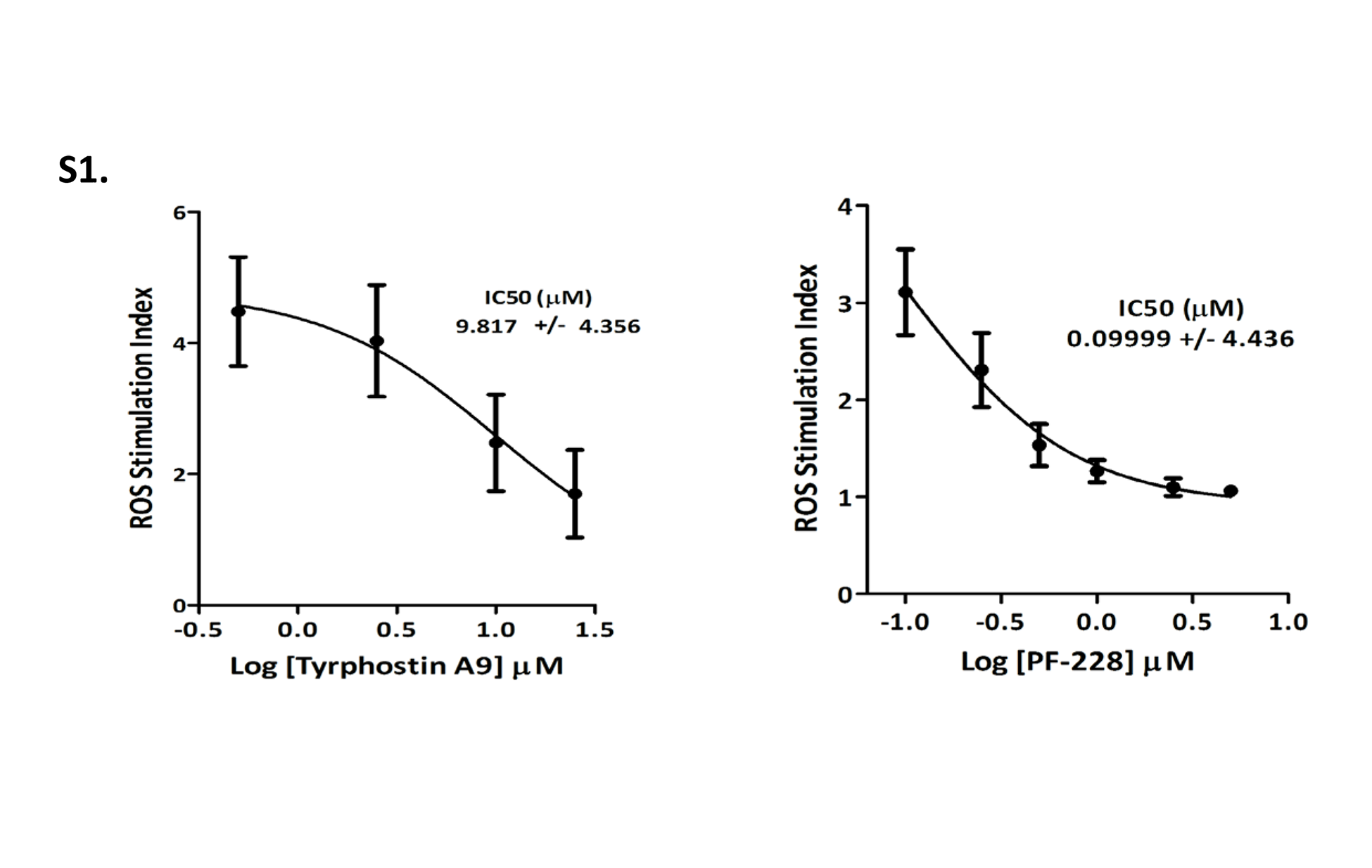

Supplement: Figure S1 — Washed human platelets (2.5×10^8^/mL) preloaded with 10 µM H2DCFDA were pre-treated with vehicle control (0.1% DMSO), Pyk2 inhibitor (0.5–25 µM Tyrphostin A9) or FAK inhibitor (0.1–5 µM PF-228), then stimulated with 1 µg/mL CRP and monitored for ROS generation. Data are plotted in Graphpad Prism as stimulation index vs log [inhibitor] µM to determine the inhibitor IC50 values. (TIF) [file pone.0113679.s001.tif]

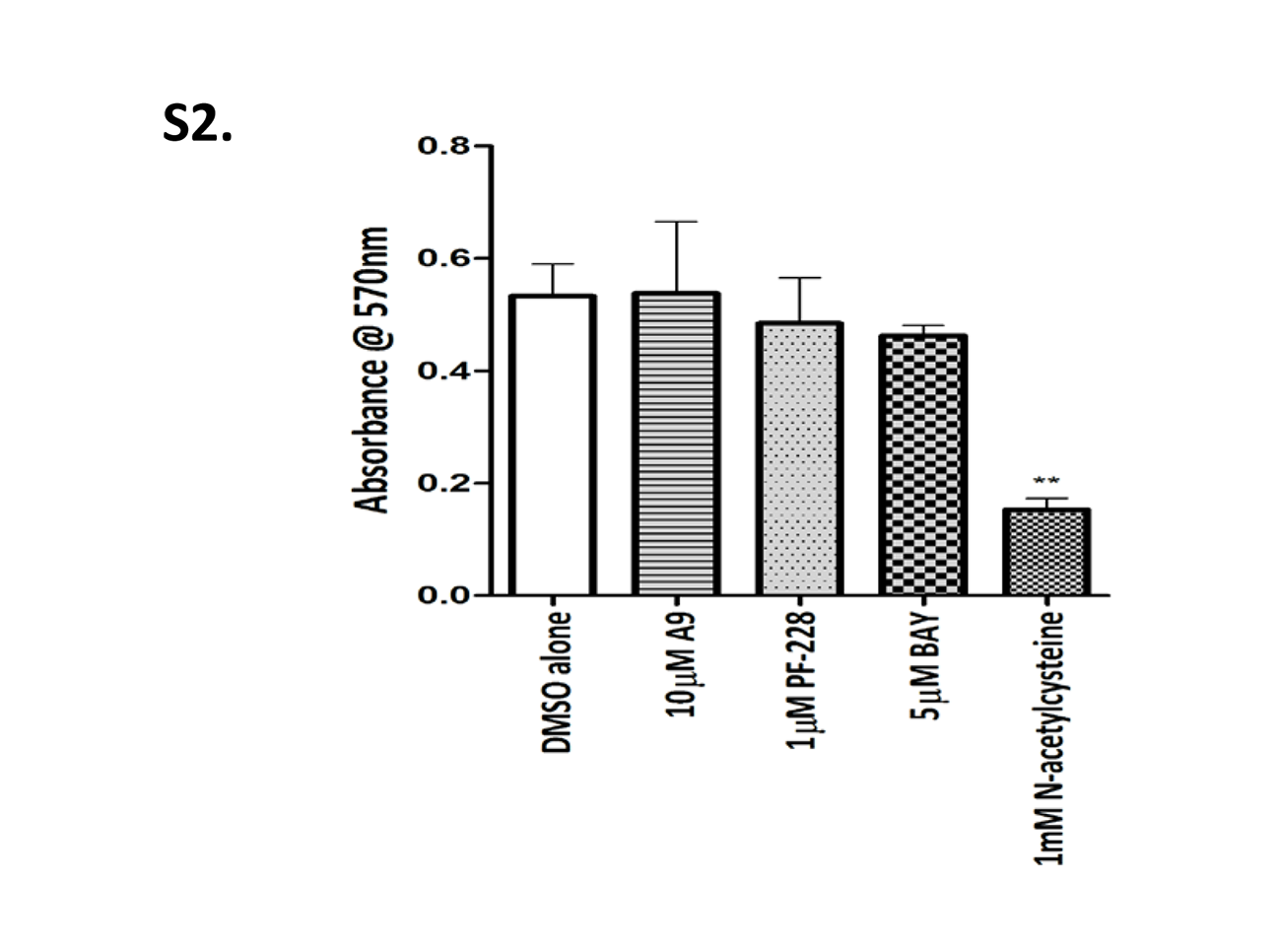

Supplement: Figure S2 — FAK and Pyk2 inhibitors do not scavenge superoxide anion. Using a cell-free superoxide anion (O2 .−) assay, pharmacological inhibitors PF-228 (1 µM), Tyrphostin A9 (10 µM) and BAY (5 µM) were tested for the capacity to scavenge ROS (N-acetylcysteine was included as positive control). Data are mean ± SEM (n = 3), **p≤0.01 vs. DMSO. (TIF) [file pone.0113679.s002.tif]

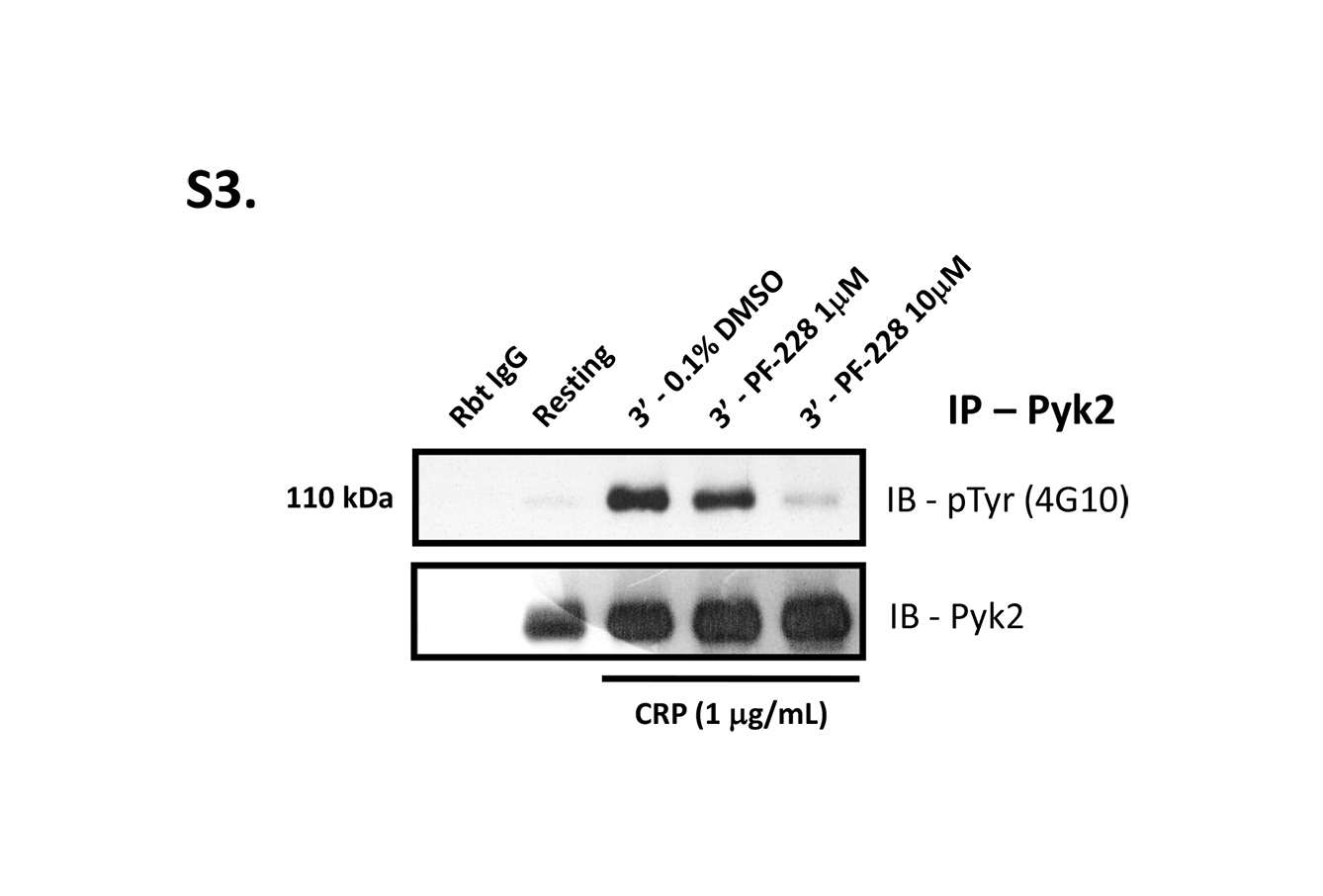

Supplement: Figure S3 — Washed human platelets pre-treated with vehicle control (0.1% DMSO) or FAK inhibitor (PF-228) at 1 µM or 10 µM were stimulated with 1 µg/mL CRP for 3 min (with stirring), immunoprecipated with anti-Pyk2 and blotted for phosphotyrosine (4G10). Blots are representative of three independent experiments. (TIF) [file pone.0113679.s003.tif]

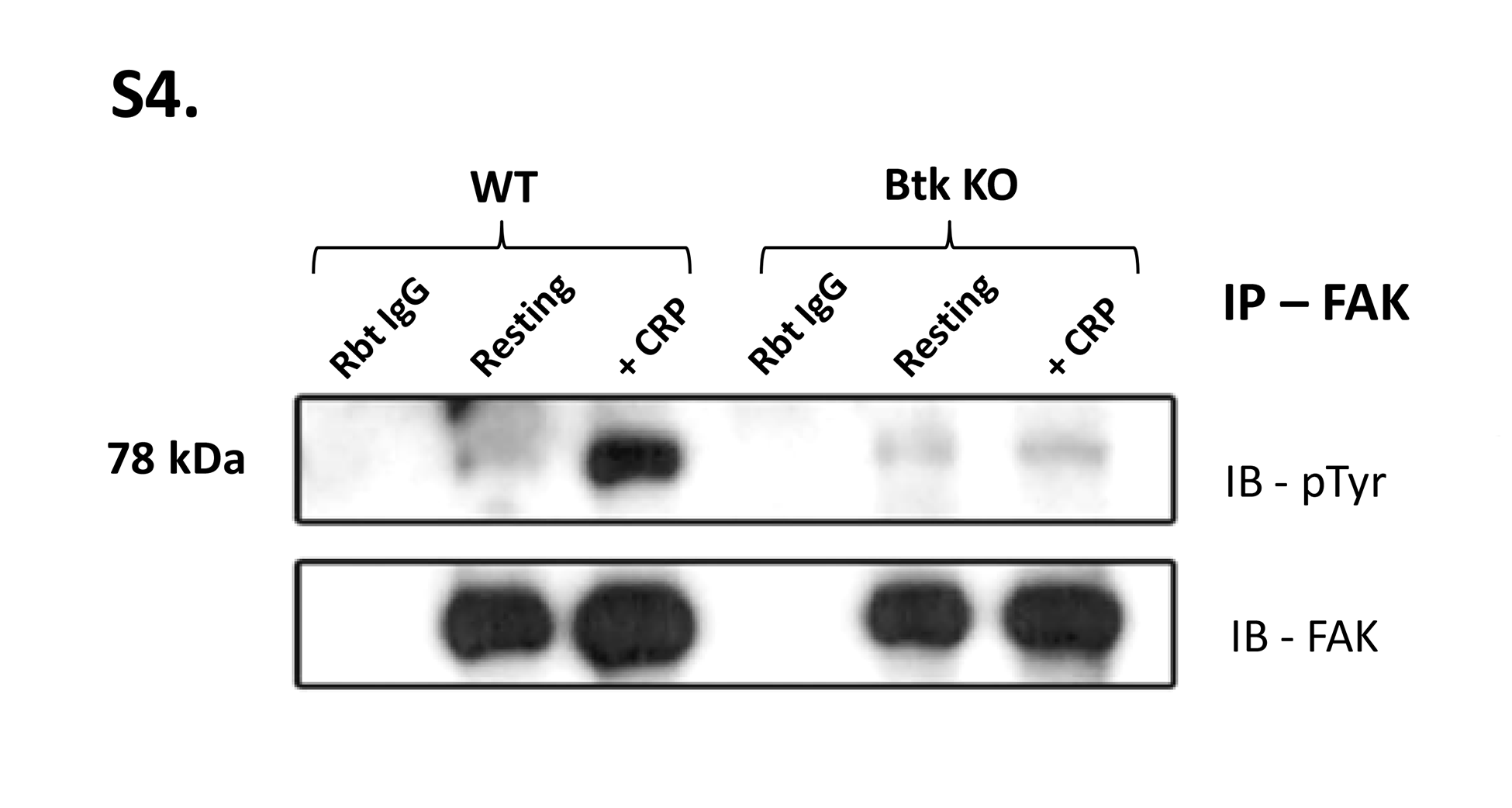

Supplement: Figure S4 — Washed platelets (1×10^9^/mL) from wildtype or Btk knockout mice were stimulated with 1 µg/mL CRP for 3 min (with stirring), lysed, immunoprecipitated with anti-FAK (4 µg), then analysed by SDS 5–20% polyacrylamide gel electrophoresis and immunoblotted for phosphotyrosine (4G10) and FAK. Blots are representative of two independent experiments. (TIF) [file pone.0113679.s004.tif]
