# Supplementary material for: Role of Focal Adhesion Tyrosine Kinases in GPVI-Dependent Platelet Activation and Reactive Oxygen Species Formation
Source: PLoS One. 2014 Nov 21;9(11):e113679. doi: 10.1371/journal.pone.0113679 (PMC4240642; doi:10.1371/journal.pone.0113679)
Supplement: Methods S1 — (DOCX) [file pone.0113679.s005.docx]

**Supplementary Methods**

*Superoxide Anion (O_2_^.-^) Assay:* This cell-free assay was used to assess potential O_2_^.-^ scavenging ability of pharmacological inhibitors. Briefly, 10 µL of PF-228, Tyrphostin A9, BAY or N-acetylcysteine (Sigma) (in 100% DMSO) was dispensed in a clear 96-well plate. 90 µL of freshly prepared alkaline DMSO (90% DMSO, 10% deionized H_2_O, 1 mM NaOH) and 20 µL of 0.25 mg/mL **Methylthiazolyldiphenyl-tetrazolium (**MTT-Sigma) were added to antagonists. After 5 min incubation, 100 µL of 100% DMSO was immediately added to terminate the reaction and absorbance (570 nm) was measured using a Wallac Victor® 1420 Multilabel counter (PerkinElmer). The decrease in the absorbance indicates the consumption of generated superoxide by antioxidants.

*Murine Bruton’s tyrosine kinase (Btk) Knockout (KO) Study*: Btk KO mice were kindly provided by Dr. Caroline Jefferies. Handling of Btk KO mice was performed in the RCSI Biological Research Facility in accordance with the Research Ethics Committee of the RCSI. The subsequent isolation of murine blood, preparation of washed platelets and immunoprecipitation procedure for quantification of tyrosine phosphorylated FAK are as described in the materials and methods.
